# Cellular Immune Dysfunction in Obstructive Sleep Apnea

**DOI:** 10.3389/fsurg.2022.890377

**Published:** 2022-07-06

**Authors:** Katharina Ludwig, Tilman Huppertz, Markus Radsak, Haralampos Gouveris

**Affiliations:** ^1^ Sleep Medicine Center, Department of Otorhinolaryngology, Medical Center of the Johannes Gutenberg-University of Mainz, Mainz, Germany; ^2^ IIIrd Department of Medicine, Medical Center of the Johannes Gutenberg-University of Mainz, Mainz, Germany

**Keywords:** obstructive sleep apnea, cellular immune system, T cells, interleukins, dendritic cells, monocytes, B cells, natural killer cells

## Abstract

Obstructive sleep apnea (OSA) is the most common sleep-related breathing disorder. Repetitive pauses in breathing during sleep cause a brief but recurrent decrease in oxygen saturation in organs and tissues (chronic intermittent tissue hypoxia). Many studies have proven a pro-inflammatory status in OSA patients. However, few reports are available on the effects of OSA on the cellular immune system, mostly focusing on single immune cell types and their subtypes. The aim of this Mini-Review is to summarize these reports, as OSA is associated with a high prevalence and comorbidities such as atherosclerosis, which are known to involve the cellular immune system.

## Introduction

Obstructive Sleep Apnea (OSA) describes a condition that is characterized by breathing interruptions during sleep, caused by a collapse of the upper airways, resulting in oxygen deficiency in the body (1). Consequences are, amongst others, sleep fragmentation and loss of the REM-phase together with daytime sleepiness (2). The severity is measured using the Apnea-Hypopnea-Index (AHI), which is calculated by dividing the number of apneas and hypopneas by the sleep duration in hours. An apnea is recorded when the airflow is reduced by 90% or more for at least 10 s, during hypopnea, airflow is either reduced by a minimum of 50% and an awakening from sleep can be observed, or the airflow is reduced by 30% or more and a desaturation of oxyhaemoglobin by at least 4% can be measured (3). Here, the following classification of severity applies: 5/h ≤ AHI < 15/h corresponds to mild OSA; 15/h ≤ AHI ≤ 30/h to moderate and an AHI > 30/h to severe OSA.

There are many epidemiological studies available regarding OSA prevalence. A common estimate is that 4% of adult men and 2% of adult women suffer from OSA (3, 4). However, a population-based study with 2121 participants found that 23.4% of women and 49.7% of men showed moderate to severe sleep-disordered breathing (5). A review of various studies allows the estimate that the prevalence in Germany is up to 60.1% (6) and supports the argument that all statistics indicate that OSA is a widespread pathology. The high discrepancy between the studies is probably due, among other things, to the dependence on study country, diagnostic method, age, previous illnesses and physical condition of the study participants. In addition, a high number of unreported cases is suspected, since patients who do not suffer acute symptoms (such as daytime sleepiness) are diagnosed less frequently.

The consequences of disturbed sleep patterns and thus OSA can be far-reaching, ranging from daytime sleepiness, declining performance, cardiovascular (e.g., myocardial infarction and stroke) and mental (e.g., depression) disorders, to cancer (7–12).

The primary effect of the airflow disruption is a low oxygen partial pressure in the blood and thus an undersupply in every organ and tissue. Usually this only lasts for a short time but occurs repeatedly during sleep and is therefore called chronic intermittent hypoxia (CIH). The resulting superoxide ions (oxygen radicals) lead to a pro-inflammatory state associated with various conditions, in particular cardiovascular disease (13–20). Given that CIH is the hallmark of OSA and promotes or induces a proinflammatory milieu, one would expect that patients with OSA should exhibit immune dysfunction.

Through nocturnal positive airway pressure (PAP) treatment, breathing interruptions can be prevented, restoring significantly the oxygenation of tissues and organs.

The aim of this narrative mini-review is to summarize the existing knowledge regarding cellular innate and adaptive immunity disorders in OSA patients. We focus on preliminary data in adults, because paediatric OSA shows different pathophysiologic features.

## Cellular Immunity and OSA

The effects of CIH on the cellular immune system in adult OSA patients have been poorly studied. Only few studies exist, most of which focus on a few specific cell types. These will be summarized in this mini-review.

### Monocytes

A recent study investigated the effect of OSA on different subtypes of monocytes (classical monocytes: CD14^++^CD16^−^, intermediate monocytes: CD14^+^CD16^+^, non-classical monocytes: CD14dim^+^CD16^+^) in patients with untreated OSA, who have an average AHI of 17.8 ± 16.96/h (21). In treatment-naïve OSA patients, it was found that there was a decrease in classical monocytes associated with an increase in CD16^+^- subtypes (intermediate, non-classical) compared to healthy subjects (21). These changes correlated strongly with patient body mass index (BMI), such that severely obese patients had the highest number of CD16^+^ monocytes. CD16^+^ subtypes are associated with increased production of tumor necrosis factor (TNF)-α and interleukin (IL) 1β (21). In addition, there was an increase in PD-L1 (programmed cell death-ligand 1) expression on monocytes from OSA patients, which correlated with the severity of the change in the monocyte population and thus BMI, AHI, and PD-L1 expression on CD4^+^ and CD8^+^ T cells. This is attributed to an increase in HIF-1α, for which CIH is causative (22–26).

In addition, a significant increase in complex formation between CD3^+^ T cells and CD14^+^ monocytes, which has been described as a potential marker of immune dysfunction (21, 27), was observed in the same study.

### T cells

Regarding T cells, several studies investigated different subpopulations. T_H17_ cells are T helper cells, are important for the adaptive immune response and are involved in the activation of neutrophil granulocytes. While Ye et al. demonstrated an increased level of T_H17_ cells in the peripheral blood of OSA patients, with an additional increase in the T_H17_/T_reg_ ratio showing a correlation with the severity of OSA (28), the concentration of T_H1_/T_H17_ cells showed no change in the study by Polasky et al. (21).

Based on the hypothesis that hypoxia-induced dysfunction of lymphocytes is involved in endothelial dysfunction in OSA, cytotoxicity of γδ T cells from OSA patients against endothelial cells was increased 2.5-fold in cell culture experiments (29). Also, the percentage of CD3^+^/γδ T cells was decreased in obese OSA patients compared with healthy subjects (30). Tissue-specific γδ T cells express a T cell receptor composed of γ- and δ-subunits (instead of α and β subunits). Their specific function and influence on specific immunological signaling pathways have not been conclusively elucidated.

Activated regulatory T cells (T_reg_) can inhibit an already established immune response by causing the secretion of certain interleukins (e.g., IL-10), which leads to a down-regulation of T effector cells. Ye et al. demonstrated in their aforementioned study that the frequency of occurrence in the peripheral blood of OSA patients was significantly decreased compared to the healthy control group, in addition to a difference between the concentration of T_reg_ cells in patients with severe OSA compared to patients with mild and moderate OSA (28). Xie et al, who detected T_reg_ cells via the markers CD4^+^CD25^+^/CD127^+^, found no changes in the concentration of T_reg_ cells in peripheral blood between a group of 39 OSA patients and a group of 33 healthy subjects (31).

In the peripheral blood of patients predominantly diagnosed with moderate and mild OSA, an increase in CD4^+^ effector T cells with a concomitant decrease in memory cell populations (effector memory and central memory T cells) was detected (21). In addition, the percentage of PD-1 positive cells proportionally to CD4^+^ and CD8^+^ cells and the intensity of PD-1 expression on CD4^+^ and CD8^+^ cells were analyzed. This was associated with an activated immune phenotype. Similarly, a slight increase in PD-L1 expression was detected on CD4^+^ and CD8^+^ T cells, although Polasky et al. reported significant differences between individual blood samples. An interesting consideration is provided by the approach to establish the relationship between the degree of change in monocyte concentration and change in T cell subpopulations. It was found that the composition of T cell subsets did not differ in samples from patients in whom there was a strong, moderate, or slight change in the distribution of monocyte subtypes. However, a significant change in PD-1 and PD-L1 expression on CD4^+^ and CD8^+^ T cells was present in the group of patients who showed the most marked change in monocyte distribution. It was concluded that the increased number of monocyte-T cell aggregates might indicate an increased interaction between cell types, leading to altered PD-1/PD-L1-mediated immune regulation. This could lead to the development of chronic inflammatory diseases (21).

Further studies on changes in the populations of CD4^+^ and CD8^+^ T cells revealed a decrease in the ratio of T helper cells and cytotoxic T cells in patients with severe OSA (32), which was attributed to a significant increase in cytotoxic (CD8^+^) T cells. An increased proportion of CD8^+^ T cells (especially CD8^+^/CD56^+^/perforin-positive ones) found in another study, may suggest involvement in inflammatory processes (33). Other authors reported a decrease in cytotoxic T cells (CD3^+^/CD8^+^) in OSA patients, which was negatively correlated with lowest oxygen saturation (31), whereas T helper cells (CD3^+^/CD4^+^) were increased, resulting in a higher CD4^+^/CD8^+^ ratio (31). Therefore, the CD4^+^/CD8^+^ ratio was positively correlated with AHI but negatively correlated with lowest oxygen saturation.

### Dendritic Cells

Antigen-presenting dendritic cells are divided into myeloid (mDCs; or conventional) and plasmacytoid (pDC) dendritic cells, with further differentiations within these subsets, e.g., mDC1 (CD1^+^) and mDC2 (CD141^+^). They have multiple functions, such as stimulation of CD4^+^ and CD8^+^ T cells, release of various cytokines, and phenotyping of T_H1_ and T_H2_ cells. Because of their low presence in peripheral blood, precise analysis of DCs is difficult, especially when subsets are considered. Two relevant studies investigated the impact of OSA on the occurrence of dendritic cells in the peripheral blood of OSA patients compared with healthy participants.

Xie et al. found no changes in dendritic cell concentration between the two cohorts (31). In contrast, a significant decrease in circulating DC in the blood of patients suffering from severe and moderate OSA was demonstrated, affecting all subsets of DCs studied (mDC1, mDC2, pDC), with mDC2 and pDC being most impaired; the concentration of these DC subtypes was at the limit of detectability in some patients (34). The decrease in mDC2 and pDC was inversely correlated with the concentration of IL-6, known to be an inhibitor of DC maturation (34).

### Natural killer and natural killer T cells

To date, there are only a few published studies on changes in natural killer cells (NK cells) and natural killer T cells (NKT cells) in OSA. In a study of 48 patients with severe OSA, it was found that both NK and NKT cells were increased in OSA patients compared to healthy control subjects. In particular, NKT cells are involved in immunological processes associated with atherosclerotic changes and hence should be considered more closely (32, 35). Contrarily, Xie et al. reported a decrease in NK and NKT cells (CD3^−^/CD16^+^CD56^+^; CD3^+^/CD16^+^CD56^+^) and a positive correlation of this decrease to the measured lowest and mean oxygen saturation, similar to the findings of Gaoatswe et al. (3, 31).

### B cells

A decrease in B cells, mediating humoral defenses, has been demonstrated in patients with severe OSA, with strong association to obesity. B cells secrete several cytokines and other cofactors (e.g., IL-10, PD-L1), which is why they have been linked to the induction of T cell apoptosis (32, 36). In contrast, Xie et al. reported an increase in CD19^+^ b lymphocytes in patients with severe and moderate OSA and explained this discrepancy by the fact that the patients recruited for their study did not have severe comorbidities, whereas the patients in the Domagala-Kulawik's study had comorbidities such as obesity, metabolic syndrome and chronic obstructive pulmonary disease (COPD), which may have influenced the expression and differentiation of immune cells such as B lymphocytes.

### Granulocytes

Of the few available studies dealing with granulocytes in OSA patients, the focus is primarily on neutrophil granulocytes. The function of granulocytes rather than the impact of OSA on their population has been predominantly studied. On the one hand, it has been reported that an increased concentration of reactive oxygen species was found in polymorphonuclear neurophilic granulocytes (37), and on the other hand, an observed reduced concentration of reactive oxygen species under hypoxic conditions is considered to be a protective mechanism against oxidative stress in OSA (38). Both experiments took place on isolated granulocytes *in vitro*. One study describes the decreasing ability of neutrohils to phagocytose bacteria and the activity of NADPH oxidase (39). In addition, an increased neurohil to lymphocyte ratio has been reported in OSA patients, which is considered a marker of inflammation (39–41). However, another study describes that no changes in neutrophil to lymphocyte ratio were observed in OSA patients (42).

### Impact of positive airway pressure (PAP) treatment on the cellular immune system of OSA patients

We have found only one study to date investigating the effects of PAP therapy on the human cellular immune system (43). Although this pilot study has reported very interesting results, it should be critically considered due to the relatively small patient cohort (25 patients) and the non-standard method of OSA-diagnosis in the respective cohort; some participants were tested by polygraphy while others by polysomnography, potentially confounding results. A redistribution in the monocyte subtypes was observed after three months (or more) of PAP therapy: there was a significant increase in the classical monocytes up to distributions similar to healthy participants with a simultaneous decrease in the intermediate and non-classical monocytes (CD16^+^).

Regarding the T cell populations analyzed (CD4^+^/CD8^+^ effector T cell groups), a significant decrease in the proportion of CD4^+^ effector T cells and an increase in effector memory cells were observed after PAP therapy. The recovery of T cell subtypes’ distribution was most pronounced in patients who showed the greatest changes within monocyte subtypes before therapy. In contrast, CD8^+^ T cells and an analysis of CD4^+^ helper T cells (T_H1_, T_H2_/T_H22_) showed no changes or differences in the proportions of individual subtypes under PAP therapy.

## Discussion

**Figure 1:**
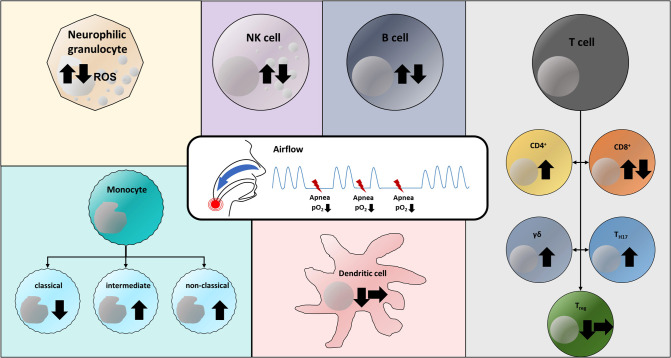
Versatile effects of obstructive sleep apnea and chronic intermittent hypoxia on the cellular immune system. Arrow up: Increase in cell population; Arrow down: Decrease in cell population; Arrow to right: No change in cell population (in OSA patients compared to respective control group). Both increased and decreased levels of reactive oxygen species (ROS) in neutrophil granulocytes have been reported (37, 38). Similarly, there are both observations on NK, NKT, and B cells describing an increase and a decrease in the respective cell population (3, 31, 32, 36, 44). CD4^+^ effector T cells have been detected at increased levels in OSA patients (21). CD8^+^ T cells were found in both increased and decreased concentrations compared with the corresponding control group (31–33). Both T_H17_ cells and γδ T cells were detected in increased concentrations in the blood of OSA patients and in cell culture experiments, respectively (28–30). Regulatory T cells have been described in reduced numbers in OSA patients (28) or at unaltered population levels (31). Studies describe that chronic intermittent hypoxia both has no effect on the population of dendritic cells (31) and leads to a decrease of these (34). Within the monocyte subpopulations, a decrease in classical monocytes and an increase in CD16^+^ subtypes (intermediate, non-classical) have been documented in OSA patients (21).

All available studies show that OSA has an impact on the cellular immune system of humans (Figure 1). A fundamental problem is that OSA is very often associated with comorbidities, such as obesity or atherosclerosis, which may also influence immunological signaling pathways and cell expression. The effects of OSA and comorbidities might interfere and therefore underlying mechanisms may be difficult to decipher. Therefore, the selection of the cohort of patients and the (healthy) control group may essentially contribute to the validity of studies in OSA patients. This is a major aspect that makes it difficult or even impossible to compare the studies described above to each other.

In addition, few data are available addressing the comparison of the same cell population or subclass of cell type, or different antibodies are used in the various studies to identify the same cell type, making it difficult to standardize the comparison of the detection intensity of the cells. In addition, deviations in the immune staining protocols or technical procedures used (timing of blood collection, available measurement systems) may also confound findings and detection. Since some subtypes, e.g. mDC2, pDC as described by Galati et al. (34), only occur in very low concentrations in peripheral blood and thus have to be partially detected at the detection limit, fluctuations around this detection limit should be considered.

It should also be noted that immune cells can influence the concentration and function of other immune cells; for instance, a decrease in B cells, which can induce T cell apoptosis under certain conditions, can influence the T cell population (32, 36).

In most studies, in addition to specific immune cells, also cytokines secreted by these immune cells, which can modulate the interaction and expression of further immune cells are investigated.

Much more studies exist on the relationship between OSA and cytokines from peripheral blood which are regulators of immune response. The majority of such studies show increasing concentrations of proinflammation cytokines in the blood and tissue (39, 45–47). In several studies IL-6, mainly secreted by monocytes, was increased in the peripheral blood of OSA patients (34, 39, 48). The increase in IL-6 has been positively correlated with the OSA severity. An association between the depletion of myeloid and plasmatic dendritic cells was also demonstrated, which correlates with the already known assumption that IL-6 is an inhibitor for the maturation of dendritic cells (34). Chronically high levels of proinflammatory cytokines may induce neuroinflammation and/or neurodegeneration (49), which is interesting because OSA is suspected to have neurodegenerative features (44, 50) and sleep disorders such as OSA occur or worsen in patients with neurodegenerative diseases, such as Parkinson's disease, Alzheimer disease or amyotrophic lateral sclerosis (51–53).

This review has some limitations. It explicitly considers only those studies that focus on the cellular immune system of the adult OSA patient. Several other studies on the cellular immune system in pediatric OSA are known, but these were purposefully not included in the review because the pathophysiology in adults and children is fundamentally different. We also refrained from discussing the many existing reports on humoral immunity in OSA to avoid losing focus on cellular immunity in OSA.

Despite all limitations and difficulties involved in the analysis of immune cells and especially the interpretation of the data, the consideration of this complex topic may provide relevant links to other related topics, such as the oncologic outcome of patients with head and neck cancer (54), which could be further investigated with markers expressed on immune cells (e.g., PD-1/PD-L1; 21, 43, 55). Considering that OSA is an inflammatory trigger and risk factor for cardiovascular disease and other comorbidities, we see great perspective opportunities to use immunological activity parameters as a surrogate for the response of OSA patients to therapies or as a starting point for the development of new therapeutic concepts.
